# Probiotic yogurt regulates gut microbiota homeostasis and alleviates hepatic steatosis and liver injury induced by high‐fat diet in golden hamsters

**DOI:** 10.1002/fsn3.3930

**Published:** 2024-01-04

**Authors:** Linwensi Zhu, Na Ying, Liuyi Hao, Ai Fu, Qinchao Ding, Feiwei Cao, Daxi Ren, Qiang Han, Songtao Li

**Affiliations:** ^1^ The First Affiliated Hospital of Zhejiang Chinese Medical University Zhejiang China; ^2^ School of Life Science Zhejiang Chinese Medical University Zhejiang China; ^3^ School of Public Health Zhejiang Chinese Medical University Hangzhou China; ^4^ Institute of Dairy Science, College of Animal Science Zhejiang University Zhejiang China; ^5^ Academy of Chinese Medical Science Zhejiang Chinese Medical University Zhejiang China

**Keywords:** gut microbiota, lipid metabolism regulation, metabolism‐related fatty liver disease, probiotic yogurt

## Abstract

This study aimed to investigate the beneficial effects of probiotic yogurt on lipid metabolism and gut microbiota in metabolic‐related fatty liver disease (MAFLD) golden hamsters fed on a high‐fat diet (HFD). The results demonstrated that probiotic yogurt significantly reversed the adverse effects caused by HFD, such as body and liver weight gain, liver steatosis and damage, sterol deposition, and oxidative stress after 8 weeks of intervention. qRT‐PCR analysis showed that golden hamsters fed HFD had upregulated genes related to adipogenesis, increased free fatty acid infiltration, and downregulated genes related to lipolysis and very low‐density lipoprotein secretion. Probiotic yogurt supplements significantly inhibited HFD‐induced changes in the expression of lipid metabolism‐related genes. Furthermore, 16S rRNA gene sequencing of the intestinal content microbiota suggested that probiotic yogurt changed the diversity and composition of the gut microbiota in HFD‐fed hamsters. Probiotic yogurt decreased the ratio of the *phyla Firmicutes/Bacteroidetes*, the relative abundance of the LPS‐producing genus *Desulfovibrio*, and bacteria involved in lipid metabolism, whereas it increased the relative abundance of short‐chain fatty acids producing bacteria in HFD‐fed hamsters. Predictive functional analysis of the microbial community showed that probiotic yogurt‐modified genes involved in LPS biosynthesis and lipid metabolism. In summary, these findings support the possibility that probiotic yogurt significantly improves HFD‐induced metabolic disorders through modulating intestinal microflora and lipid metabolism and effectively regulating the occurrence and development of MAFLD. Therefore, probiotic yogurt supplementation may serve as an effective nutrition strategy for the treatment of patients with MAFLD clinically.

## Graphical abstract

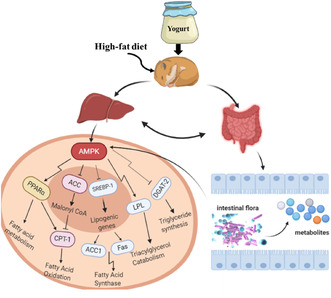

## 1 INTRODUCTION

Metabolic‐related fatty liver disease (MAFLD) has become a widespread chronic liver disease worldwide, and its global prevalence is estimated to be about 25%, which is rising rapidly (Maurice & Manousou, 2018). It has become a major disease affecting the health of residents, bringing a heavy financial burden to society and patients' families, and represented as a major public health problem that needs to be resolved urgently (Vilar‐Gomez & Chalasani, 2018). Although increasing evidence has shown that MAFLD is closely related to many factors such as diet, genetics, metabolism and insulin resistance syndrome, dyslipidemia, and changes in gut microbiota, the exact mechanism underlying its pathogenesis is still not fully elucidated (Suárez et al., 2017), and there is still no effective and precise treatment/medication that has been reported for treating the MAFLD clinically. Currently, MAFLD is considered to be reversed through lifestyle modification, such as weight loss, diet changes, and increasing physical exercise (Romero‐Gómez et al., 2017; Safari & Gérard, 2019). Therefore, finding safety foods with high prevention and/or therapeutic efficacy becomes very urgent.

The intimate relationship between the incidence of MAFLD and the intestinal micro‐ecological environment disorder has been identified, with the deepening of intestinal micro‐ecology research and deepening understanding of the pathological physiology of the “gut–liver axis” (Kirpich et al., 2015). Based on this, some researchers have proposed a “multiple strike theory” including the theory of gut microbiota imbalance and related metabolic endotoxemia translocation, which makes intestinal microbiome become a hotspot in MAFLD research and makes the gut become a promising target organ in MAFLD research (Leung et al., 2016). This conclusion has been confirmed by the research which has found that obese mice could obtain more energy from food through via their intestinal microbes, and by transplanting the gut microbiota of obese mice to sterile mice, increasing body weight and liver fat content in sterile mice were observed, indicating that imbalance of gut microbiota plays a detrimental role in the development of MAFLD (Caussy et al., 2018). Meanwhile, gut microbiota may play an important role in the pathological process of MAFLD as an important part of the gut–liver axis, and it inhibits the level of oxidative stress, reduces liver inflammation, and accelerates lipid metabolism. Currently, probiotic yogurt, a combination of prebiotics and probiotics, is the most used gut microbiota homeostasis regulator clinically and plays a positive effect on the balance of the gut microbiome (Farzanegi et al., 2019). Numerous studies have demonstrated that probiotics improve gut microbiome composition, suggesting that synbiotics affect gut microbiome that can promote the treatment of MAFLD by improving the main factors that cause the disease (Mokhtari et al., 2017; Woodhouse et al., 2018). However, the molecular mechanism and target of improvement of MAFLD by specific lactic acid bacteria are not yet clear. Therefore, it is of particular interest to elucidate the molecular mechanisms of probiotic yogurt involved in MAFLD pathogenesis.

Increasing evidence, both in vitro and in vivo, suggests that multiple factors can regulate the development of MAFLD, including the associated gene expression of triacylglycerol (TG) and free fatty acid (FFA) (Alves‐Bezerra & Cohen, 2017). Adenosine 5′‐monophosphate (AMP)‐activated protein kinase (AMPK) is an indispensable protein kinase in the body and participates in various metabolic processes, especially glycolipids metabolism (Gao et al., 2018). AMPK activation reduces the incidence of MAFLD through inhibiting fat production and increasing fatty acid oxidation in the liver. AMPK also promotes mitochondrial functional integrity in adipose tissue (Smith et al., 2016). As a downstream protein of AMPK signaling molecule, sterol regulatory element‐binding protein (SREBP) directly participates in regulating the expression of fatty acid, TG synthesis, and glucose metabolism‐related enzyme genes Acetyl‐Co A Carboxylase (ACC) and Fatty Acid Synthase (FAS) (Mun et al., 2019). Furthermore, Peroxisome Proliferator‐activated receptors α (PPAR‐α) regulate the transcription of most genes involved in fatty acid oxidation and TG transport in the liver (Samuel & Shulman, 2018). PPAR‐α reduces the exchange of neutral lipids between high‐density lipoprotein and very low‐density lipoprotein, promotes the removal of low‐density lipoprotein, increases the activity of lipoprotein esterase, and promotes the decomposition of TG (Liss & Finck, 2017). The AMPK signaling pathway reduces FFA synthesis and promotes FFA oxidation to reduce lipid deposition in peripheral tissues by inhibiting ACC expression (Park et al., 2019).

The improvement of yogurt in MAFLD has been proven, but there is no published report that fully clarifies how yogurt can synergistically enhance or regulate a series of genes by activating AMPK signaling molecules and enhancing the expression of related genes (such as PPAR‐α) and its receptors (Wu et al., 2015). The transcription of factors related to lipid metabolism and how to protect the liver by regulating the gut microbiome is not yet clear. In addition, choosing an ideal animal model with typical MAFLD characteristics and similar to humans is also the key point to study the efficacy of yogurt. In the present study, researchers tend to use golden hamsters to establish the MAFLD model, because its lipid metabolism characteristics are closer to humans and more sensitive to cholesterol and lipids, with short modeling time and low cost (Bhathena et al., 2011; Briand et al., 2012). It is of great significance for the establishment of accurate MAFLD model and related drug development to explore the technical methods suitable for studying the occurrence, development, and evolution of MAFLD of golden hamster. The purpose of this study is to demonstrate the preventive effects of probiotic bacteria in yogurt on liver steatosis, sterol deposition, oxidative stress, and gut microbiota regulation in HFD‐induced obese MAFLD rat models, and to further explore its potential mechanisms on gene regulation involved in FFA and TG metabolisms.

## 2 MATERIALS AND METHODS

### 2.1 Ethics statement

All protocols have been approved by the Medical Ethics Committee of Zhejiang Chinese Medicine University (Hangzhou, China) and implemented in accordance with the National Institutes of Health regulations on the care and use of animals in research.

### 2.2 Animal care and experimental protocol

Male golden hamster (*Mesocricetus auratus*, Vital River Laboratory Animal Technology Co., Ltd., Beijing, China, SPF grade, 4 weeks old, 132 ± 4 g) was maintained in the specific pathogen‐free (SPF) animal room. Free diet, controlled room at 22°C ± 3°C temperature, at 55 ± 15% humidity, and the 12‐hour circadian rhythm (8:00–20:00) was adopted.

### 2.3 Experimental animal grouping

The golden hamsters were adapted for 1 week before the experiment. Weigh the golden hamsters in random groups (*n* = 10 per group): normal feed control group (ND), high‐fat feed group (HFD), milk feeding group (Milk), yogurt high‐dose group (Yogurt‐H), and yogurt low‐dose group (Yogurt‐L). Animals were caged in groups according to different feeding methods. The general feed for the ND group was formulated as follows: 18% crude protein, 4% crude fat, 1.25% choline, and 5% crude fiber, vitamins, minerals, amino acids, etc. The other groups of golden hamsters were fed high fat and cholesterol feed to make animal models of non‐alcoholic fatty liver under the feed formula as follows: 79% basic feed, 10% fat, 10% egg yolk powder, and 1% cholesterol. At the same time, at 10 a.m. every day, the Milk group received 2 mL of pure milk, the Yogurt‐H group received 2 mL of yogurt (provided by the cooperative company Zhejiang Yiming Food Co., Ltd., Lactic acid bacteria contained in yogurt is a patented strain of bacteria, number: CNCMI‐2980, content ≥13 billion cfu/100 mL), and the Yogurt‐L group used 1 mL yogurt + 1 mL ddH_2_O that was given by intragastric administration. Both the ND and HFD groups were given the same volume of ddH_2_O as a placebo intragastric administration in the same way and fed for 8 weeks. Food intake and body weight were measured daily and weekly, respectively.

### 2.4 Sample collection

Golden hamsters in each group were fasted 12 h before the end of the treatment. After anesthetized with 10% chloral hydrate (w/w) intraperitoneal injection, blood was collected by abdominal aortic puncture, and serum was separated and cryopreserved for subsequent analysis; Liver, beige fat, white fat, and brown adipose tissue were separated, collected, and weighed. The intestinal contents of fresh colon tissue are collected and used to detect intestinal gut microbes.

### 2.5 Serum test

Aortic blood from each group of golden hamsters was taken, and serum was collected by centrifugation. The contents of total cholesterol (TC), triacylglycerol (TG), high‐density lipoprotein (HDL), and low‐density lipoprotein (LDL) in serum were analyzed using a fully automatic biochemical analyzer. Taking approximately, liver tissue was added in pre‐chilled physiological saline to the ice bath at a ratio of 1:9, homogenized at high speed. The supernatant after centrifugation supernatant was collected from each sample the content of malondialdehyde (MDA) and superoxide dismutase (SOD) was detected by ELISA detection kit (Shibayagi Co., Ltd., Gunma, Japan).

### 2.6 Histopathological testing

Approximately, 3 mm of liver tissue from each group was taken and fixed in 4% neutral formaldehyde. After 48 h of fixation, it was dehydrated, embedded, sliced, and stained with hematoxylin and hematoxylin–eosin (HE). At the same time, the fresh liver part was quickly frozen, and the cryostat section was cut and stained with Oil Red O. The liver pathological changes were observed under the Olympus optical microscope.

### 2.7 mRNA expression level detection

Liver tissues were frozen at −80°C. TRIzol reagent (Invitrogen, CA) was used to extract total RNA from liver tissues, digested at 37°C for 5 min, reverse transcribed to obtain cDNA, and the expression level was detected by fluorescence quantitative PCR. Primer5 and DNAman software design, ACC, CPT‐1, DGAT‐2, LPL, PPAR α, and SREBP‐1 primers, using β‐actin as internal reference (primer sequence information see Table 1): PCR reaction conditions: 94°C, 1 min 95°C 10 s, 58°C 10 s, and 72°C 10 s (40 cycles).

**TABLE 1:** Primer sequence for quantitative real‐time PCR.

Gene	Sense(5′‐3′)	Antisense(5′‐3′)
LPL	CAGCTGGGCCTAACTTTGAG	CCTCTCTGCAATCACACGAA
PPAR‐alpha	GAAACTGCCGACCTCAAAT	CAGCATTCCGTCTTTGTTC
CPT‐1	ATCTTCCAGTTGGGCTACG	GCAGGTCCACATCATTCG
SREBP‐1c	AGACAAACTGCCCATCCATC	CACCCTCCATAGCCACATCT
ACC	TGTGAGCCTGAGGAATAGCA	GAGCAATCCACCATCACTCA
DGAT‐2	TCTCAGCCCTCCAAGACATC	ATGCCAGCCAGGTGAAGTAG

### 2.8 Protein expression level detection

Western blot method was used for protein detection. An appropriate amount of liver tissue was fully milled under liquid nitrogen environment, RIPI lysate was lysed on ice for 10 min, centrifuged at 1200 rpm/ min for 5 min, the supernatant was taken, and protein concentration was determined by BCA. Same amount of total protein (60 μg) was used from each sample for 20% SDS‐PAGE electrophoresis, PVDF membrane transfer, 5% skimmed milk powder, and block at 4°C overnight, and primary antibody dilution (1:500) was added and incubated at room temperature for 2 h and then washed the membrane three times with Tris–HCl buffered salt solution + Tween (TBST). Horseradish peroxidase‐labeled secondary antibody (1:1000) was incubated at room temperature for 2 h, and TBST washed the membrane three times, developed by the DAB method, and imaged by the gel fiber camera system. The experiment was repeated at least three times, showing representative blots.

### 2.9 16S rDNA detection of structural differences in the gut microbiome

Gut contents of each group were collected in a sterile EP tube and stored at −80°C prior to use. For DNA extraction, total genomic DNA was extracted from the collected specimens using commercial DNA extraction kit DNA concentration was verified using, and the extracted DNA was stored at −80°C prior to use. Total DNA was sent to Huada Gene Technology Co., Ltd. for high‐throughput detection of the 16S rDNA gene V4 region. The results were analyzed by bioinformatics, and the representative sequence of OUT was compared with the database. Use Mothur software (version 1.33.3) for Alpha diversity analysis (Chao, Ace and other species richness statistics, Shannon, Simpson and other species diversity statistics), VENN diagram, dilution curve, and Beta diversity analysis ((un) Weighted UniFrac analysis). For species‐based beta diversity analysis, heatmap drawing, multi‐sample similarity tree drawing, and principal component analysis (PCA) R language (version 3.2.3) were used.

### 2.10 Statistical methods

SPSS 21.0 was used for statistical analysis of the obtained data. The count data were represented by [*n* (%)], the X2 test was used, the measurement data were represented by (Mean ± SD), and the t‐test was used. *p* < .05 indicates the statistical significance differences.

## 3 RESULT

### 3.1 Effect of yogurt on body weight and fat content

**FIGURE 1:**
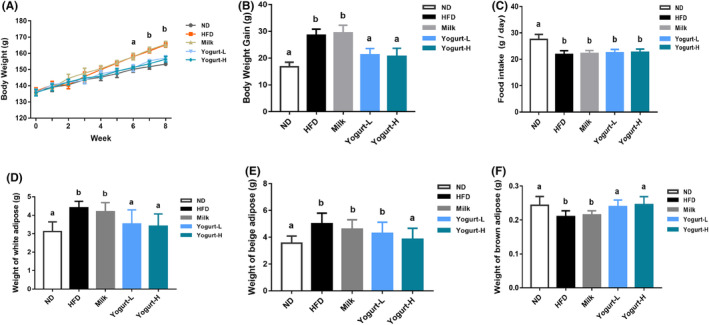
HFD‐induced obese NAFLD rat model. The body weight gain and biochemical indexes are presented as follows: (A) body weight gain, (B) body weight gain, (C) food intake, (D)weight of white adipose, (E) weight of beige adipose, and (F) weight of brown adipose. Values are means 6 SEM (NFD, *n* = 13; HFD, *n* = 70). The values with different superscripts are significantly different at *p <* .05.

No significant difference was found in body weight of golden hamsters at the beginning of the experiment among all groups (Figure 1A). The body weight, liver weight, and liver index of golden hamsters were significantly increased after 8 weeks of high‐fat diet (HFD) feeding (*p* < .05). Yogurt administration significantly reduced HFD and milk‐induced weight gain, respectively (Figure 1B). No difference was observed in food intake among four groups fed the high‐fat diet (*p* > .05) (Figure 1C). Compared with control group, HFD induced a significant increase in the white fat content of the golden hamsters (Figure 1D), whereas it was significantly reversed by high intake of yogurt. The high intake of yogurt significantly relieved the accumulation of fat in the body but increased the brown fat content, making the two indicators no significant difference between yogurt‐H group and ND group (Figure 1E,F).

### 3.2 Serum biochemical indicators

**FIGURE 2:**
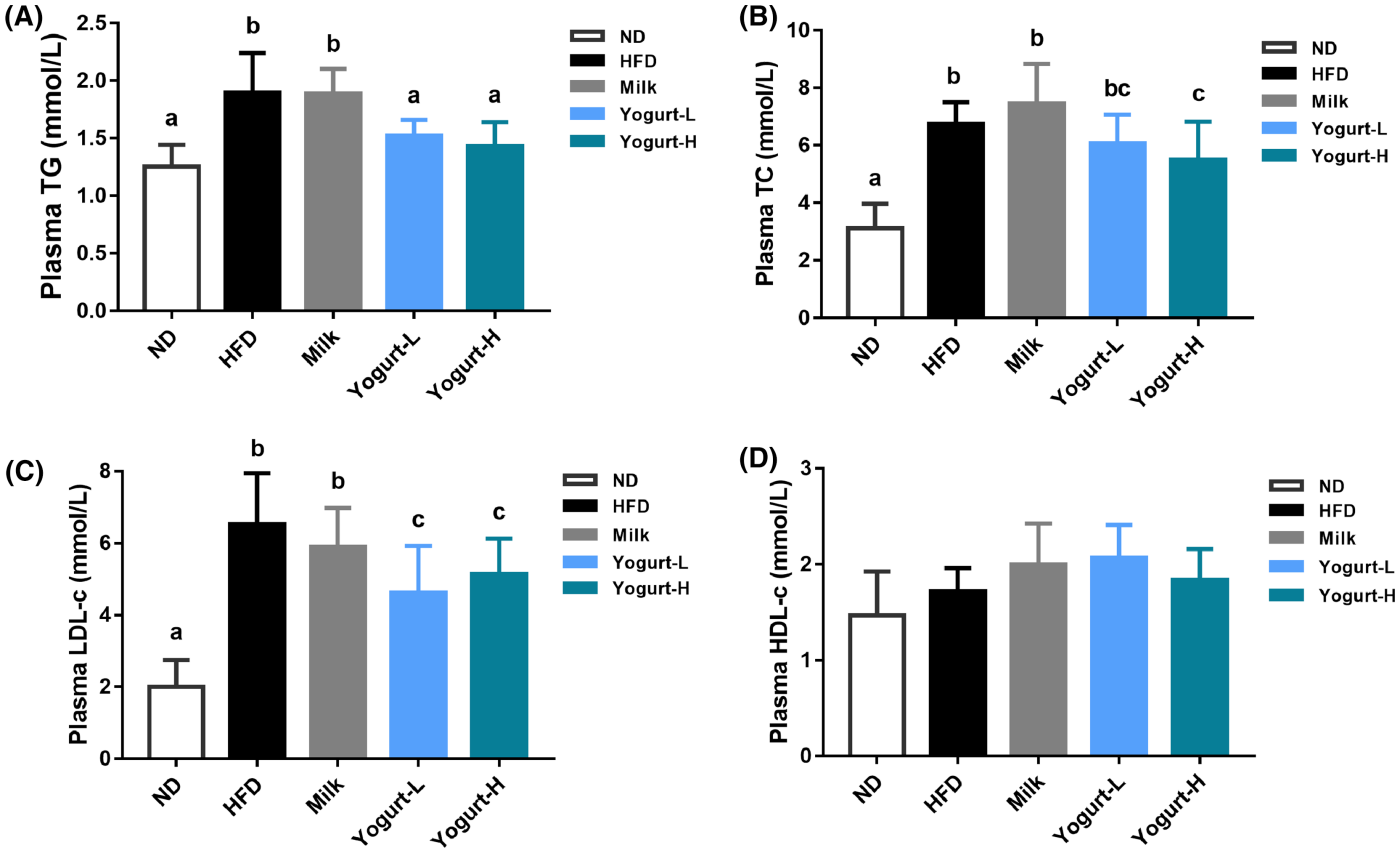
Effects of yogurt on the serum TG, LDL‐c, and HDL‐c. The biochemical indexes are presented as follows: (A) plasma TG, (B) plasma TC, (C) plasma LDL‐c, (D) plasma HDL‐c. Groups annotated with “a, b, c” differed significantly with *p* < .05 as determined by one‐way ANOVA and a Tukey's test. All the groups contain 10 animals (*n* = 10).

Yogurt rescued the adverse changes of serum biochemical indicators caused by HFD. Compared with ND golden hamsters, the levels of plasma TG, TC, and LDL‐C were significantly increased in HFD golden hamsters (Figure 2A,C), while HDL‐C levels were comparable (Figure 2D). Both the high and low supplementary doses of yogurt significantly reduced the high expression of serum TG, TC, and LDL‐C induced by HFD in a dose‐dependent manner (Figure 2B).

### 3.3 Inhibition of liver damage caused by HFD

**FIGURE 3:**
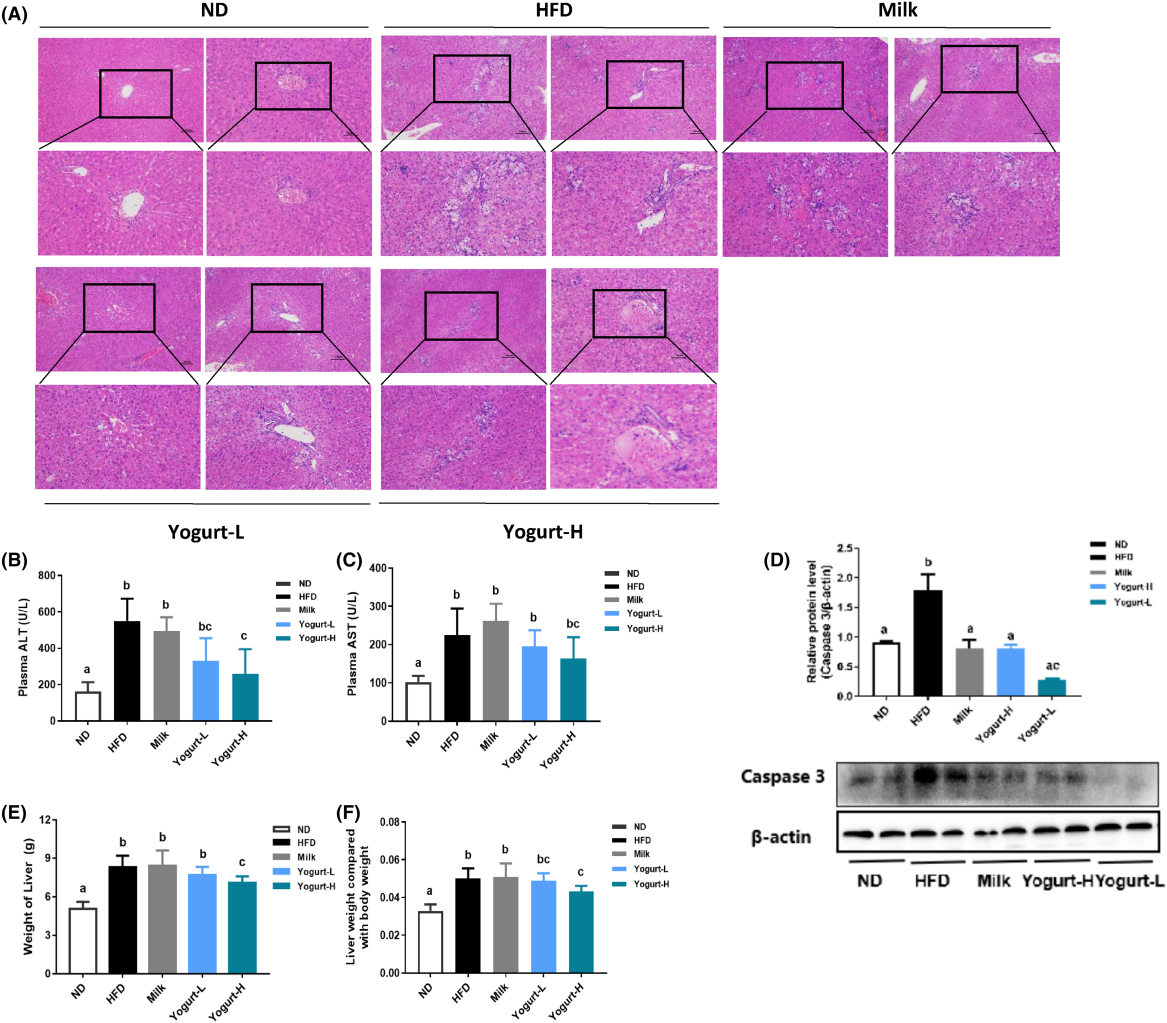
Effects of yogurt on the liver damage indicators. The representative photographs and biochemical indexes are presented as follows: (A) H&E staining photomicrographs of the liver section (1006), (B) plasma ALT, (C) plasma AST, (D) relative protein Caspase 3/β‐Actin, (E) weight of liver, and (F) liver weight compared with body weight. Groups annotated with a, b, c differed significantly with *p* < .05 as determined by one‐way ANOVA and a Tukey's test. All the groups contain 10 animals (*n* = 10).

HE staining analysis showed no obvious liver histopathological changes in the ND group, the leaflet structure in the slices was normal, there were no obvious pathological changes such as infiltration of inflammatory cells and no obvious fibrosis in the manifold area, the liver cells were neatly arranged, the cytoplasm was uniform, and the shape was regular. No vacuoles were seen in the cytoplasm (Figure 3A). Long‐term HFD significantly induced the accumulation of TC and TG in the liver of golden hamsters, promoted the formation of MAFLD, and increased the level of circulating liver enzymes (including AST and ALT) (Figure 3B,C), promoting the development of liver injury. HFD also showed greater effect on the liver weight and liver index of golden hamsters. Compared with other high‐fat diet groups, Yogurt‐H group significantly slowed down the increase in liver weight and liver index (Figure 3E,F) and ameliorated liver lipid deposition. Apoptosis of liver cells is the key marker of MAFLD (Kanda et al., 2018). The Caspases (Cysteine‐requiring. Aspartate Proteases) enzyme in hepatocytes is the main apoptotic effector and plays a key role in the signal transduction system (Al Mamun et al., 2020; Zhao & Sun, 2020). Caspase 3 enzyme is a critical terminal cleavage enzyme (Sakasai‐Sakai et al., 2017). It plays a decisive role in the progression of apoptosis (Choudhary et al., 2015), and its activation occurs in the early apoptosis of cells. HFD could activate the activity of Caspase through phosphorylation, induce DNA degradation (Figure 3D), and ultimately lead to liver cell damage and apoptosis.

### 3.4 Liver steatosis

**FIGURE 4:**
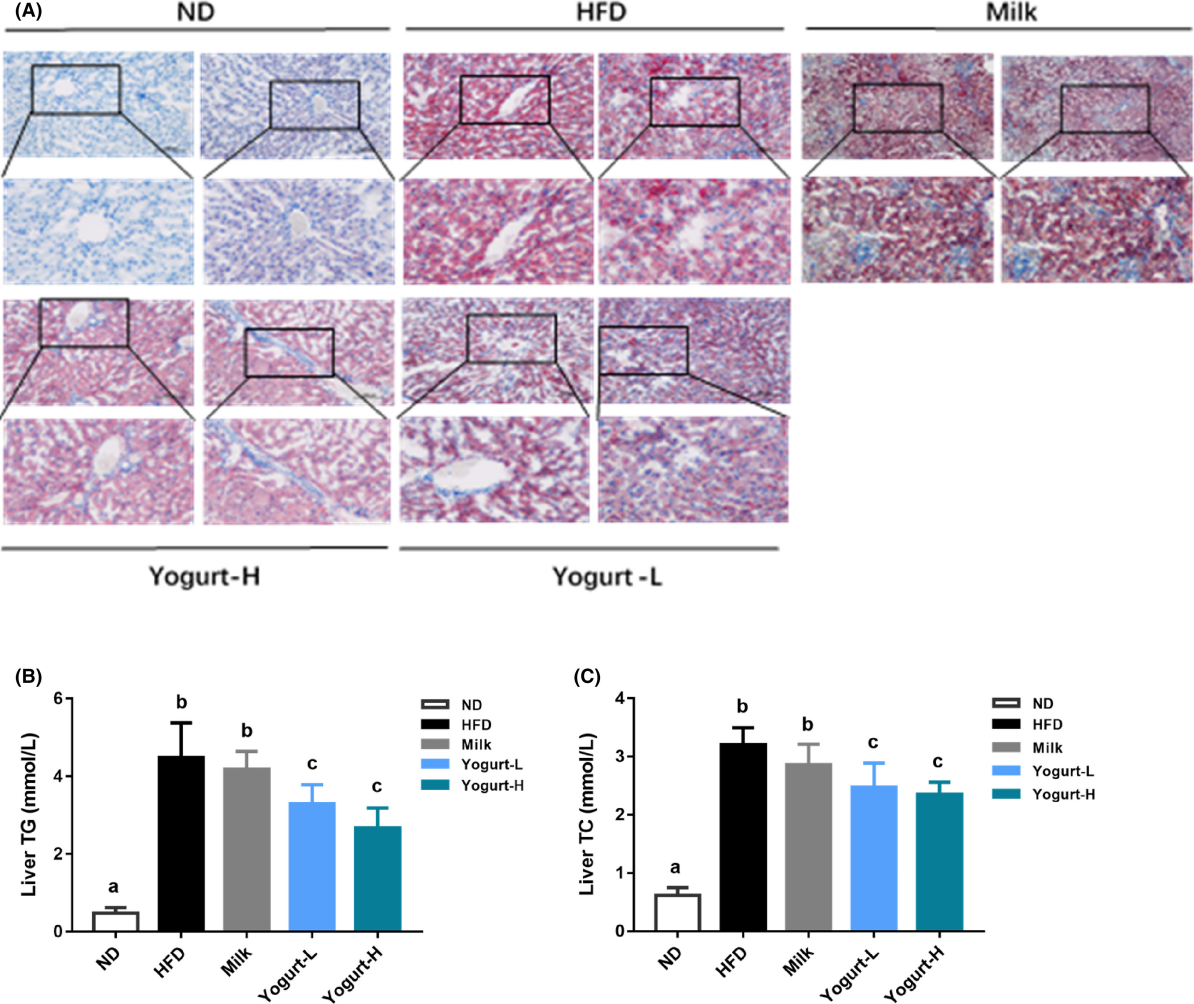
Effects of yogurt on the liver. The representative photographs and biochemical indexes are presented as follows: (A) H&E staining photomicrographs of the liver section, (B) liver TG, (C) liver TC. Groups annotated with a, b, c differed significantly with *p* < .05 as determined by one‐way ANOVA and a Tukey's test. All the groups contain 10 animals (*n* = 10).

Oil red O analysis showed that no obvious lipid accumulation was observed in the liver lobule (Figure 4A). The long‐term high‐fat diet‐feeding model of golden hamsters induced large‐scale hepatic steatosis in the liver, and pathological changes were observed, including inflammatory cell infiltration in the manifold area and inflammatory bodies in hepatic lobule. Formation of hepatocytes, balloon‐like changes in hepatocytes, pale staining of cytoplasm and cytoplasm, visible obvious lipid droplets of different sizes, shift of the nucleus: oil red O visible red lipid droplets in liver lobule under staining (Figure 4A), and yogurt supplementation significantly reversed the above adverse changes induced by HFD. Through experimental comparison, we found that the hepatic TG and TC levels in HFD‐fed golden hamsters were significantly higher than that of golden hamsters fed with normal diet (Figure 4B,C). The intervention of yogurt significantly reduced the TG and TC accumulation in the liver.

### 3.5 Changes in the structure of gut microbiota

**FIGURE 5:**
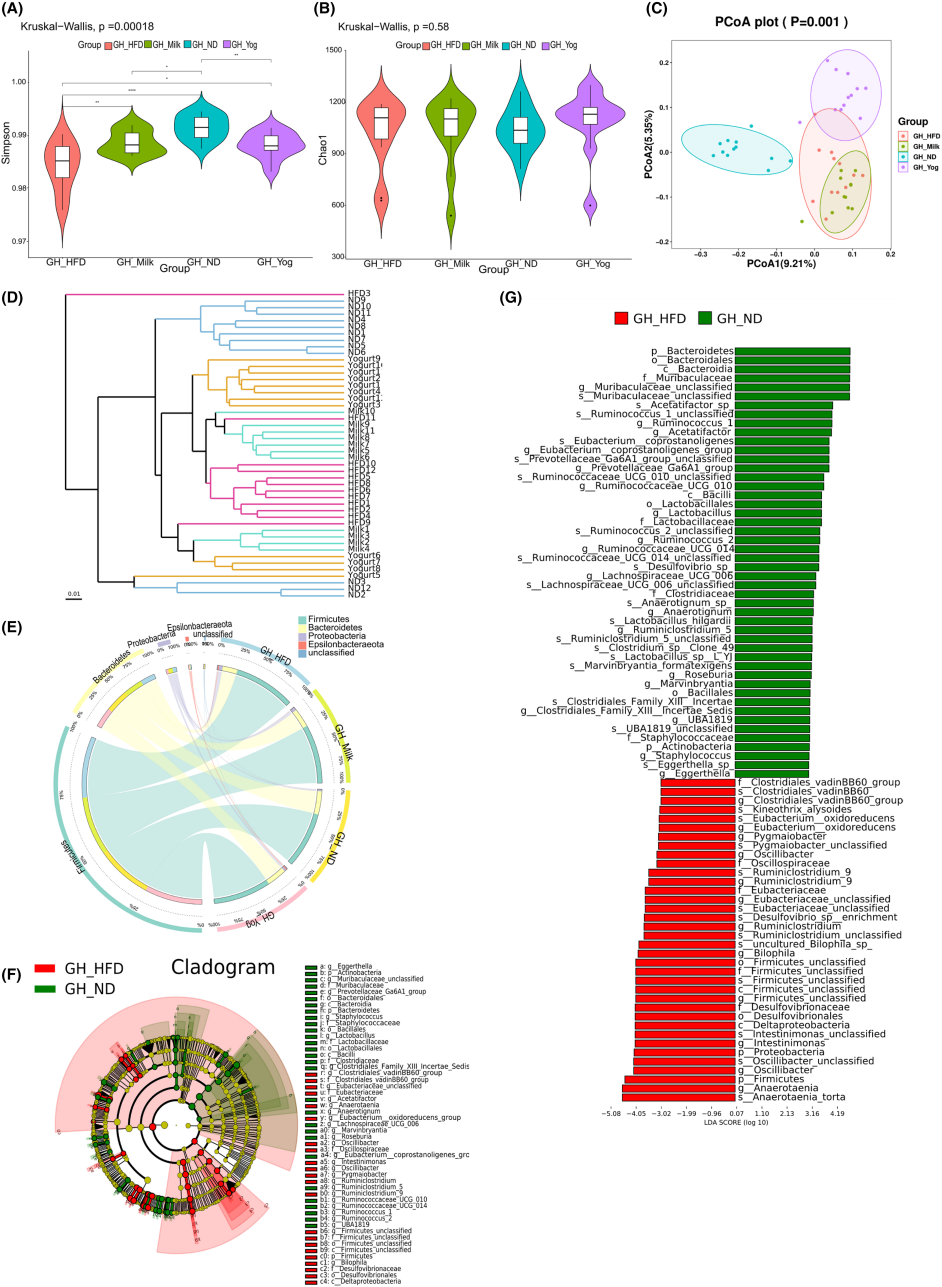
Effects of yogurt on the structure of gut microbiota. (A) the abundance of gut microbiota. (B) the diversity of gut microbiota. (C) PCoA analysis. (D) UPGMA analysis. (E) circos circle diagram of the horizontal level. (F) Cladogram analysis. (G) LEfSe species difference analysis. All the groups contain five samples (*n* = 5).

Although HFD and yogurt administration did not significantly affect the abundance of gut microbiota among all groups (*p* > .05), the diversity of gut microbiota was significantly reduced (*p* < .01) (Figure 5A,B). PCoA analysis showed that the distance between the three groups of the high‐fat diet and the ND group was large, and the sample was dispersed as a whole, indicating that the high‐fat diet caused the overall composition of the sample to be different, with significant differences. The samples in the HFD and milk groups were relatively concentrated. After the intervention in the yogurt group, the colony body tended to move away from the HFD group, and there was a significant change (Figure 5C). Similarly, unweighted pair‐group method with arithmetic means (UPGMA) analysis further proved that the samples of the HFD diet and the general diet group were distributed in different branches on the cluster tree, and the samples of the HFD diet were clustered and distributed, indicating that the two groups of samples were significantly different in evolution (Figure 5D). To further compare the differences caused by HFD and yogurt interventions, the gut microbiota structure of each group of golden hamsters was analyzed based on the gate level. Circos circle diagram of the horizontal level of the door shows that *Firmicutes, Bacteroidetes, Proteobacteria*, and *Epsilonbacteraeota* dominate the four groups of golden hamster gut microbiota. Compared with ND, the proportion of thick‐walled bacteria (such as *Firmicutes*) significantly increased in the gut microbiota of golden hamsters in the high‐fat feeding group, while the proportion of *Bacteroidetes* decreased. Compared with the HFD group, the proportion of *Firmicutes* in the gut microbiota was decreased in the yogurt group, and the proportion of *Bacteroides* in the phylum *Bacteroides* was increased in golden hamsters (Figure 5E). LEfSe species difference analysis found that the *Ruminococcaceae* abundance value in the gut microbiota of the yogurt group and the ND group increased significantly compared to the golden hamsters of the HFD group, indicating that the intervention of the milkshake application adjusted the gut microbiota to make it and the groups are closer (Figure 5F). In addition, *p‐Bacteroidetes*, *o‐Bacteroidales*, *c‐Bacteroidia*, and other *Bacteroidetes* belonging to the genus of *Bacteroidetes* showed significant differences in the yogurt group (Figure 5G).

### 3.6 Regulation of TG‐ and FFA‐related gene expression

**FIGURE 6:**
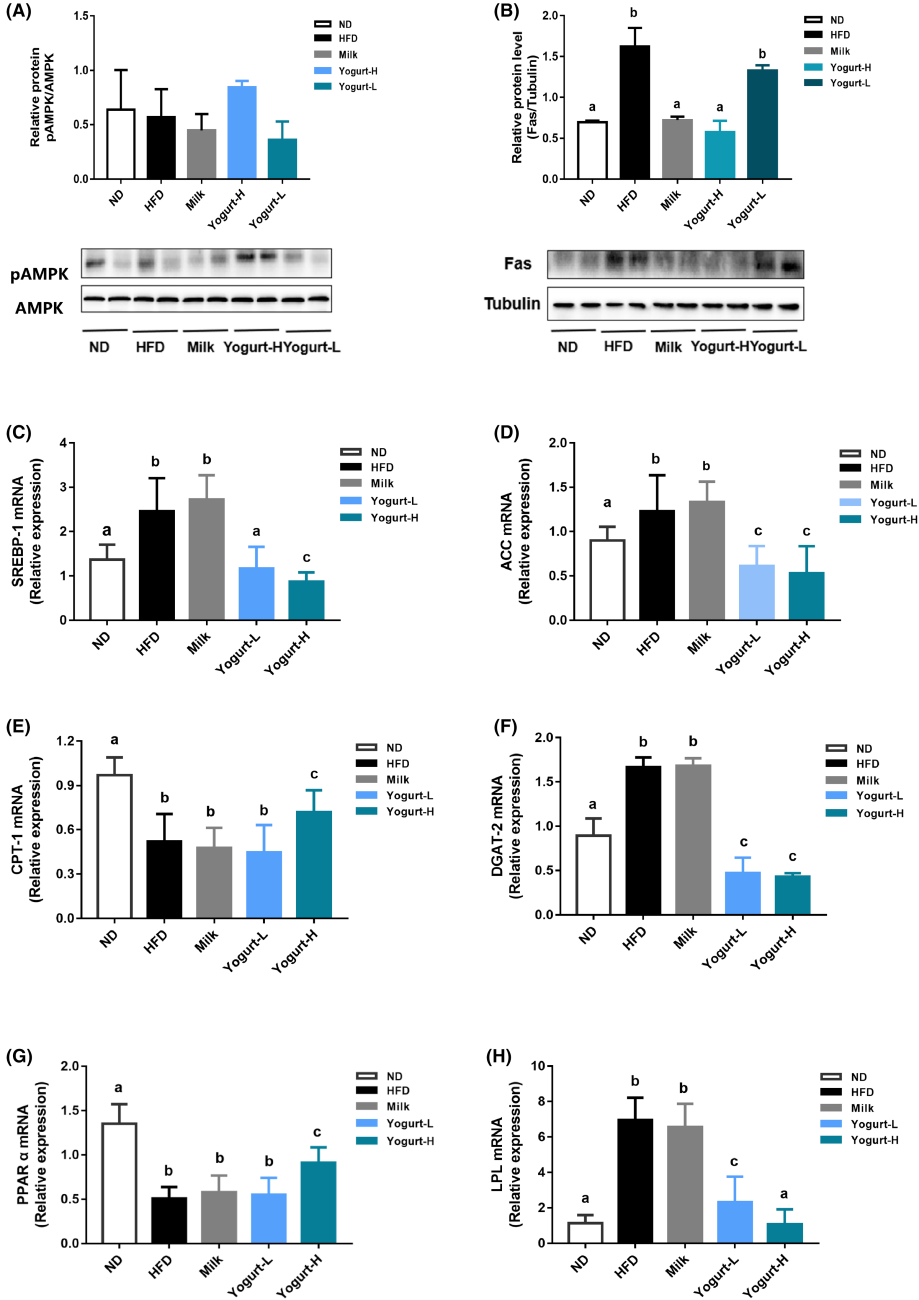
Effects of yogurt on TG‐ and FFA‐related gene expression. The representative photographs and biochemical indexes are presented as follows: (A) The levels of protein of p‐AMPK, (B) the levels of protein of Fas, (C) the levels of mRNA of SREBP‐1, (D) the levels of mRNA of ACC, (E) the levels of mRNA of CPT‐1, (F) the levels of mRNA of DGAT‐2, (G) the levels of mRNA of PPAR, (H) the levels of mRNA of LPL. Groups annotated with “a, b, c” differed significantly with *p* < .05 as determined by one‐way ANOVA and a Tukey's test. All the groups contain 10 animals (*n* = 10).

HFD‐induced disorder lipid metabolism‐related gene expression is closely related to the pathogenesis of MAFLD (Wu & Ni, 2020). Yogurt administration significantly reversed HFD‐decreased phosphorylated AMP‐activated protein kinase (AMPK) protein levels (Figure 6A), indicating that the intervention of the mixed group could activate AMPK. High‐fat diet increased the expressions of hepatic FAS and ACC, and two downstream targets of SREBP‐1 were significantly reduced by yogurt treatment (Figure 6B–D). Conversely, the mRNA expressions of PPAR α and CPT‐1 related to FA metabolism and oxidation were upregulated by yogurt treatment (Figure 6E–G). DGAT‐2, the main enzyme for TG synthesis, was upregulated by HFD and significantly downregulated by yogurt supplementation on mRNA level. LPL, a key enzyme related to the hydrolysis and metabolism of triglycerides (Péterfy et al., 2007), was significantly reduced by yogurt treatment (Figure 6H), suggesting that the release of free fatty acids in body was reduced.

### 3.7 Hepatocyte oxidative damage stress

**FIGURE 7:**
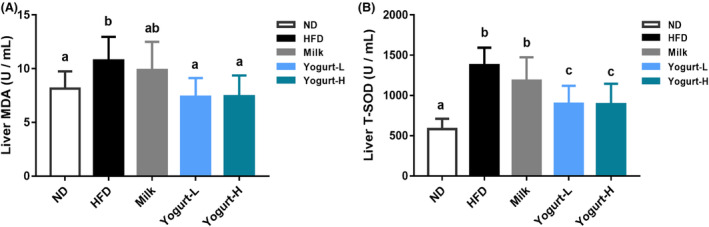
Effects of yogurt on the hepatocyte oxidative damage stress. (A) liver MDA, (B) liver T‐SOD. Groups annotated with “a, b, c” differed significantly with *p* < .05 as determined by one‐way ANOVA and a Tukey's test. All the groups contain 10 animals (*n* = 10).

The increase in liver T‐SOD and MDA levels eventually leads to the increased oxidative stress. The intervention of yogurt reduced the increase in the above indicators and reversed the liver damage caused by HFD in golden hamsters (Figure 7A,B).

## 4 DISCUSSION

Based on golden hamster MAFLD model, we revealed that yogurt supplementation could ameliorate the liver damage of MAFLD by modulating the gut microbiome dysbiosis, metabolism disorders, and oxidative stress (Jahn et al., 2019).

The dietary factors have been considered an essential role in the development of MAFLD (Perdomo et al., 2019; Watzinger et al., 2020). A healthy diet lifestyle has become the main way to improve the prognosis of MAFLD (Romero‐Gómez et al., 2017). The use of high‐fat‐diet feeding to establish an animal model of MAFLD has become one of the basic measures to explore the pathogenesis of MAFLD and develop effective treatments (Im et al., 2021). Present study showed that after 8 weeks of high‐fat‐diet feeding, the body weight growth rate of golden hamsters was significantly higher than that in the normal group. Moreover, H&E and Oil Red O staining analysis indicated that the hepatic histopathological changes were observed in HFD‐fed animal, suggesting that MAFLD golden hamster model was successfully generated.

The high prevalence of MAFLD among obese people indicates the importance of treating obesity as one of the most important factors related to the disease (Polyzos et al., 2019). Yogurt is a well‐known healthy food, which plays a protective role in the development of obesity and MAFLD (Chen et al., 2019). The basic research on the “anti‐obesity” effect of probiotics is mostly probiotics from the genus Lactobacillus, and the other is the genus Bifidobacterium, which also provides new methods for preventing obesity (Li et al., 2016; Million et al., 2012). Several studies have shown that probiotic yogurt can affect the gut microbiome balance, which in turn affects the body's caloric intake, gut absorption, and energy balance (Kok & Hutkins, 2018; Wen & Duffy, 2017) In our study, we administered high‐dose and low‐dose gavage probiotic yogurt, respectively, and by reducing food/energy intake, we found that the body weight and liver weight were decreased, and the brown fat level was increased. The decrease in white adipose and serum LDL‐C indicates that yogurt reduces fat production, increases lipolysis and fat oxidation, reduces body weight, and reverses fatty liver disease caused by obesity.

Oxidative stress plays an important role in the pathogenesis of MAFLD (Spahis et al., 2017). According to the “two‐hit” theory of MAFLD pathogenesis (Day & James, 1998), oxidative stress is involved in the “second‐hit.” Furthermore, oxidative stress participates in the pathogenesis of MAFLD through mechanisms such as increased reactive oxygen species (ROS), lipid peroxidation, Kupffer cell activation, and mitochondrial dysfunction (Bessone et al., 2019). MAFLD patients displayed higher (hepatic or circulation) levels of ROS and lipid peroxidation products than healthy people. In addition, various studies have shown that the level of lipid peroxidation products is directly related to the severity of MAFLD (Albano et al., 2005; Bellanti et al., 2017). Endogenous ethanol and its derived compounds (acetaldehyde and acetic acid) are produced in the gut microbiome and increase ROS production by Kupffer cells and hepatic stellate cells (HSCs) (Pendyala et al., 2011). By modulating the gut microbiota to use symbiotics, the production of ROS and lipid peroxidation product formation can be ameliorated, which improve MAFLD development by reducing pathogen‐related oxidative stress (Safari & Gérard, 2019). In our study, the yogurt treatment showed significant antioxidant capacity by improving hepatic steatosis and inhibited the production of reactive oxygen metabolites and cytokines that damage pancreatic cells. Thereby, it reversed the increase of liver transaminase (including ALT and AST) and MDA, which were indexes of liver cell damage caused by high‐fat diet, reduced the oxidative stress response of the liver, and promoted the improvement of the patient's condition. Some randomized controlled trials have also demonstrated the effect of yogurt on oxidative stress in patients with MAFLD.

Yogurt is mainly composed of prebiotics and probiotics (Sáez‐Lara et al., 2016). Prebiotics are indigestible carbohydrates. By changing the composition and activity of gut microbiome, it can play a positive role in maintaining the health of the host. Probiotics are also known as non‐pathogenic live microorganisms when administered in adequate amounts confer a health benefit on the host. Several studies have also shown that gut microbiota status is closely related to the pathogenesis of MAFLD (Wang et al., 2016). The possible mechanisms include the release of lipopolysaccharide, increased ethanol production, and activation of inflammatory cytokines in luminal epithelial cells and kupffer cells (Kazankov & Jørgensen, 2019; Leung et al., 2016; Schoeler & Caesar, 2019). The abundances of *Bacteroidetes* and *Pachyphyta* are generally considered to be closely related to obesity and are the targets of effective treatment by regulating the flora (Mouzaki et al., 2013). In our study, PCoA analysis and UPGMA analysis suggested that 8 weeks of yogurt intervention could improve the gut microbiome structure of golden hamsters fed by an HFD. The Bacteroides phylum in HFD group was significantly increased, and the thick‐walled phylum was significantly decreased. After yogurt intervention, the ratio of the Bacteroides phylum and the thick‐walled phylum was decreased and the structure tended to be similar in the ND group. The gut microbiome structure of MAFLD rats caused by diet is disordered. Analysis between LEfSe groups found that *Ruminococcaceae* significantly increased in the yogurt and ND group, and *Ruminococcaceae* included the important butyrate‐producing *C. tenderum*. Butyrate is a short‐chain fatty acid produced by resistant starch, dietary fiber, and low‐digestible polysaccharides through fermentation of the microbiome and the distal small intestine (Sivaprakasam et al., 2016). As shown in our study, butyrate‐producing probiotics could reduce MAFLD progression in rats. Zhou Da et al. also confirmed that sodium butyrate reduced HFD‐induced steatohepatitis in mice by improving gut microbiota dysbiosis and gut barrier functions (Zhou & Fan, 2019). With the introduction of the concept of “gut–liver axis,” more and more evidence show that there is a close relationship between gut microbiome and liver diseases. The gut microbiome can increase the amount of food energy extracted and promote the decomposition of adipose tissue and reduce deposition by activating corresponding lipid regulatory genes (Le Roy et al., 2013). Use Spearman correlation analysis to find the genus of gut microbiota associated with MAFLD disease indicators. Based on gate‐level analysis, the results showed that *Firmicutes* were significantly positively correlated with plasma ALT, LPL mRNA, DGAT‐2 mRNA, the weight of liver (*p* < .01), *Proteobacteria* showed significantly positively correlated with LPL mRNA, body weight gain, weight of liver, liver TG, liver TC (*p* < .01), *Bacteroidetes* showed significantly negatively correlated with LPL mRNA, plasma ALT, Plasma AST, DGAT‐2 mRNA (*p* < .01). Actinobacteria showed significantly negatively correlation with liver TG, liver TC, plasma HDL‐C, plasma LDL‐C (*p* < .01), while showed significantly positively correlation with CPT‐1 mRNA (*p* < .01). Based on the description of the above results, we could speculate that probiotic yogurt could improve the gut microflora structure disorder of MAFLD in golden hamsters, making it tend to the gut microflora structure of ND group golden hamsters. Probiotic yogurt regulates the occurrence and development of MAFLD through the gut microbiome, which may be an effective and promising nutrition strategy to prevent it clinically.

This study has proved that probiotic yogurt could effectively reduce body weight and reduce lipid accumulation by inhibiting lipid synthesis in the liver. Probiotic yogurt also promotes hepatic fat metabolism and reduces oxidative stress (Fardet & Rock, 2018). Nonetheless, the fatty acid metabolism process in the liver is more complicated, mainly including fat mobilization, fatty acid activation, fatty acid and triacylglycerol synthesis, β‐oxidation, and fatty acid transport (Nguyen et al., 2008). These processes involve a variety of key enzymes (such as ACC, FAS) and transcription factors (such as SREBP‐1, PPAR‐α) (Chen et al., 2017). The reduction of liver triglycerides and the increase of fatty acid oxidation‐related gene levels lead to an increase in total energy expenditure. AMPK is called “receptor of cell energy balance,” which is widely distributed in tissues with strong energy metabolism, such as liver, fat, skeletal muscle, etc. It participates in the process of MAFLD and plays an important role in regulating fatty acid metabolism (Garcia et al., 2019). In our study, yogurt activated the expression of p‐AMPK at the protein level. It was speculated that the short‐chain fatty acid (SCFA) and SCFAs produced by probiotic bacteria and induced by probiotic bacteria during yogurt fermentation are potential AMPK agonists. AMPK‐mediated activation can trigger the expression of peroxisome proliferator‐activated receptor‐gamma coactivator 1α (PGC‐1α), thereby controlling the transcriptional activation of transcription factors involved in the regulation of fatty acid metabolism, such as peroxisomal proliferation biological activation receptor PPAR‐α (Cantó & Auwerx, 2009). PPAR‐α is a subtype of PPARs nuclear receptor supergene family, which mainly promotes the transmembrane transport of fatty acids by inducing the expression of fatty acid transporter (FATP) and fatty acid translocase (FAT/CD36) and high‐dose intake yogurt significantly activates PPAR‐α mRNA expression (Wierzbicki et al., 2009). By stimulating PPAR‐α to regulate the transcription of the rate‐limiting enzyme gene CPT‐1, it plays a key role in regulating liver lipid metabolism, so it is a widely accepted potential therapeutic target (Xu et al., 2017).

SREBP‐1c is highly expressed in adipocytes and liver tissues, and causes disturbance of glucose and lipid metabolism, increases in fat volume, and lipid accumulation in the liver and other non‐adipose tissues (Payne et al., 2009). It regulated target genes including ACC, FAS as well as other related fatty acid synthesis and sugar metabolism genes (Fang et al., 2019). This experimental study showed that the yogurt group could significantly increase the mRNA and protein expressions of ACC and PPAR‐α, and transport fatty acids through fatty acid transporters and transposases, thereby increasing the decomposition of triacylglycerol. Furthermore, the mRNA and protein expressions of hepatic SREBP‐1c and FAS were significantly reduced by yogurt supplementation. By inhibiting the expression of glycolipids, fat accumulation was reduced. In addition, the target gene SREBP‐1c and FAS reduces fatty acid absorption by inhibiting its fatty acid synthesis. For another target gene, ACC may increase due to the inhibition of SREBP‐1c or other signaling pathways, and phosphorylation promotes the oxidation of fatty acids to regulate liver steatosis. Taking together, yogurt intake reduces the expression of SREBP‐1c and FAS in the liver of golden hamsters, and inhibits fatty acid absorption and fat synthesis in the liver by up‐regulating the expression of PPAR‐α and ACC in the liver which further promotes the oxidation of fatty acids, and the decomposition of fat in the liver.

Treatment of early MAFLD by reducing TG synthesis is considered to be an effective strategy (Kang et al., 2021). DGAT2 is an important enzyme that controls the rate of TG synthesis (Chitraju et al., 2019; Yen et al., 2008). The effect of yogurt on inhibiting its activity may lead to a decrease in the rate of TG synthesis, and the possibility of intracellular apolipoprotein B migrating into the endoplasmic reticulum, resulting in increased degradation of apolipoprotein B. Multiple studies have shown that DGAT2 deficiency caused a significant reduction of fatty acids as the fatty acids are an important part of TG synthesis. After the mouse was knocked out of the DGAT2 gene, almost no TG could be detected in the tissues tested, and plasma glucose, TG, and FFA levels were reduced by 70%–90% (Stone et al., 2004); therefore, the lack of DGAT‐2 significantly reduced the TG synthesis and overexpression of DGAT‐2 led to the accumulation of a large number of lipid droplets in the cytosol. However, our present results confirmed that yogurt decreased the DGAT2 mRNA expression level in the liver by inhibiting lipid deposition and ultimately improved symptoms of MAFLD. Yogurt enters the body through oral administration and considers its recognized gut microbiome regulation, so we speculate that yogurt may play a role in the prevention and treatment of MAFLD by regulating the gut microbiome structure, protecting the gut mucosal barrier and other mechanisms.

## 5 CONCLUSION

In summary, for the first time, we deeply evaluated the intervention effect of probiotic yogurt in the pathogenesis of MAFLD, through the gut microflora and lipid metabolism genes in the golden hamster model. Our results indicated that probiotic yogurt might be effective in improving the structure of the intestinal flora, regulating the related genes of the important AMPK pathway in lipid metabolism, inhibiting lipid synthesis in the liver, promoting fat metabolism, reducing oxidative stress, etc., which could be effective to reduce body weight, reduce lipid accumulation, and improve the occurrence and development of MAFLD. Taken together, the use of dietary strategies targeting the gut microbiota has emerged as an additional tool to control metabolic diseases.

## AUTHOR CONTRIBUTIONS

**Linwensi Zhu:** Data curation (equal); methodology (equal); writing – original draft (equal). **Na Ying:** Data curation (equal); software (equal); writing – original draft (lead). **Liuyi Hao:** Investigation (equal); resources (equal); software (equal). **Ai Fu:** Formal analysis (equal); investigation (equal); writing – review and editing (equal). **Qinchao Ding:** Methodology (equal); project administration (equal); validation (equal). **Feiwei Cao** and **Daxi Ren:** Data curation (equal); formal analysis (equal); software (equal). **Qiang Han** and **Songtao Li:** Conceptualization (lead); methodology (lead); supervision (lead).

## FUNDING INFORMATION

This work was supported by the National Natural Science Foundations of China (No. 81973041), Zhejiang Natural Science Foundations for Distinguished Young Scholars (No. LR20H260001), Zhejiang Chinese Medicine University School‐level Scientific Research Fund Project (No. 2020ZR07, No. 2021JKJNTZ004A), and Special Support Program for High Level Talents in Zhejiang Province (No. ZJWR0308092).

## CONFLICT OF INTEREST STATEMENT

The authors have declared no conflict of interest.

## ETHICS STATEMENT

This study was approved by Ethics Committee of Zhejiang Chinese Medicine University (No. IACUC‐20211129‐12).

## Data Availability

The data that support the findings of this study are available on request from the corresponding author. The data are not publicly available due to privacy or ethical restrictions.
